# Investigating the Interplay Between Sleep, Anxiety, and Depression in Chronic Kidney Disease Patients: Implications for Mental Health

**DOI:** 10.3390/healthcare13030294

**Published:** 2025-01-31

**Authors:** Reynita Saguban, Asmaa Mohamed Ali AlAbd, Evalyn Rondilla, Joyce Buta, Salwa Ali Marzouk, Richard Maestrado, Chandrakala Sankarapandian, Sameer A. Alkubati, Romeo Mostoles, Salman Amish Alshammari, Maha Sanat Alrashidi, Analita Gonzales, Grace Ann Lagura, Ferdinand Gonzales

**Affiliations:** 1 College of Nursing, University of Hail, Hail 55473, Saudi Arabia; reynsaguban@gmail.com (R.S.); a.alabd@uoh.edu.sa (A.M.A.A.); e.rondilla@uoh.edu.sa (E.R.); j.buta@uoh.edu.sa (J.B.); s.marzouk@uoh.edu.sa (S.A.M.); r.maestrado@uoh.edu.sa (R.M.); alkubatisa@yahoo.com (S.A.A.); maha-838@hotmail.com (M.S.A.); g.lagura@uoh.com (G.A.L.); 2 Medical-Surgical Nursing Department, College of Nursing, Princess Nourah bint Abdulrahman University, P.O. Box 84428, Riyadh 11671, Saudi Arabia; csshenbagathammal@pnu.edu.sa; 3 Emergency Nursing Department, King Khalid Hospital, Hail 55421, Saudi Arabia; slman2024@hotmail.com; 4 Faculty of Nursing, University of Tabuk, Tabuk 71491, Saudi Arabia; a_gonzales@ut.edu.sa; 5 College of Nursing, King Khalid University, Abha 62521, Saudi Arabia; f.gonzales@kku.edu.sa

**Keywords:** prevalence, sleep disorders, depression, anxiety, chronic kidney disease

## Abstract

Background/Objectives: This study aims to determine the level of anxiety, depression, and sleep disturbances in patients with chronic kidney disease, as well as the interactions between the three comorbidities, and the implications it has for health practitioners. Methods: A descriptive cross-sectional study, following the Strengthening the Reporting of Observational Studies in Epidemiology (STROBE) guidelines, was implemented whereby 179 patients with chronic kidney disease attending a government hospital were recruited to this study. Results: Our results showed that 65.9% of the participants had the metrics of severe anxiety while 34.1 percent had moderate anxiety. Further, it was reported that more than half 51.4% of participants had poor sleep quality. The levels of depression reported by the patients were roughly 40.2% minimal depression, 29.6% mild, 21.2% moderate, and 8.9% depression that was severe. There was a significant correlation between mental health disease together with sociodemographic variables such as gender, marital status, educational status, and nationality (*p* < 0.05). Cut points of those aged 26–35 found younger patients experiencing higher anxiety levels (cut point (AOR = 2.792; *p* = 0.021)), and they also had poorer sleep quality (AOR = 0.403; *p* = 0.020). Conclusion: Our findings illustrate the importance of more frequent early mental health measures and strategies that help patients with chronic kidney diseases. The main study limitation was the cross-sectional design, which allows for correlational but not causal statements to be made. We address a gap in the literature with our results by noting specific demographic characteristics that are associated with poor mental health in chronic kidney disease populations and discuss practical and policy recommendations aimed at enhancing the mental well-being of chronic kidney disease patients.

## 1. Introduction

Chronic kidney disease (CKD) is considered one of the leading health challenges of the present world due to its adverse effects, including mental depression, anxiety, and sleep issues [1]. Recently, it has been recognized that many patients having chronic kidney disorders manifest signs of severe anxiety or depression; this has further necessitated new research on redefining these issues as pre/post-disorders of CKD [2]. For patients with depression and anxiety, insomnia and other sleep disorders are known problems that have been studied, but the interplay between depression, anxiety, and sleep is less studied. In the existing literature, most studies focus on depression, repeatedly use it as a monovariable, and pay negligible attention to the bi-directional relationship sleep and anxiety or depression has [3,4]. This gap in the body of knowledge necessitates a change in the understanding of CKD patients.

There are several different areas from which the neurobiological links between sleep, anxiety, and depression stem. It has been suggested that sleep disturbances activate the stress response, which in turn may cause or worsen anxiety or depression, and this forms a vicious cycle that limits treatment [5]. For example, there is evidence that failure to shift circadian rhythm increases the chances of developing CKD, which means that disturbances in sleep quality are more than an outcome of CKD, but at times enhance the condition in the case it develops [6]. In addition, researchers suggest that obstructive sleep disorders become more common with the deterioration of CKD, and this makes the care of these patients all the more complex [7]. This highlights the importance of considering sleep as well as mental health problems to reduce the overall burden on patients. On the other hand, most available works do not seem to consider the impact of sociodemographic variables. Studies indicate that differences in age, gender, social standing status, and education may impact the quality of sleep and mental health status in patients with CKD [8,9]. For instance, the older population of CKD patients seems to have high levels of anxiety and depression, which on the other hand greatly lowers their quality of sleep [10,11]. This emphasizes that the perspective of these sociodemographic factors should also be included in studies about the mental health issues of patients with CKD as they may add an essential understanding of the multi-faceted nature of these patients’ lives.

While there has been an increase in the literature in this area, there is still a lack of understanding regarding the relationship between sleep, anxiety, and depression in CKD patients. That is, most previous research has dealt with either anxiety or depression on its own, and most of the time, the reciprocal relationships between the factors and sleep have been overlooked [12]. Furthermore, there are few, if any, integrative programs that take into consideration these intertwined problems simultaneously. As much as some psychosocial measures have been effective in reducing anxiety and depression in chronic disease patients, their use in CKD patients is still minimal [13]. This means that the use of comprehensive approaches that holistically tackle these issues is lacking.

The relevance of this research rests upon the perspectives it brings to the understanding of the interactions between sleep, anxiety, and depression disorders with CKD, which is a global health problem. With this, it is intended to contribute to the existing literature on these comorbidities and the mechanisms that underlie them in enhancing the understanding of the interrelationship between sleep and mental health. Such insights will help to design therapies that not only target patients’ psychological problems but also aim at improving CKD patients’ general lives and their clinical results. It has been noted that sleep disorder treatment occurs amongst CKD populations and most times leads to adverse outcomes; the results of this study will thus serve as a guide for policies and best treatment practices in most if not all healthcare systems on how to manage patients and prevent adverse results following a holistic approach to the treatment of CKD. Hence, this study aims to investigate the complex interplay between sleep, anxiety, and depression in CKD patients.

## 2. Methods

### 2.1. Design

This study used a cross-sectional design. It strictly adhered to the Strengthening the Reporting of Observational Studies in Epidemiology (STROBE) guidelines, promoting transparency and methodological rigor.

### 2.2. Participants/Setting

Participants were 179 patients diagnosed with CKD taken via convenience sampling. This was in an effort to minimize bias by including detailed demographic and clinical data, inviting all eligible patients to participate, and ensuring confidentiality and clear instructions for self-reporting. All participants met established criteria for CKD (e.g., eGFR levels), were willing and able to provide informed written consent, and were able to understand and complete the study questionnaires. Patients younger than 18 years were excluded. Participants were chosen based on practical considerations such as the availability of eligible patients, the resources available for this study, and the desired level of precision. The participants were recruited in single large urban government hospital situated in Hail City, Saudi Arabia, as it is noted for its capacity to deal with a substantial number of patients with CKD, enabling effective recruitment of participants. While it is acknowledged that such a strategy may restrict generalization, the investigators took great precautions to limit potential confounding variables by obtaining rich demographic and clinical characteristic data, including comorbid status and medications, for baseline measures. To avoid any sort of bias, all patients who met the criteria were requested to take part in the trial. In this study, self-reporting was the key method of gathering data through questionnaires and may have contributed to an element of bias in reporting. Nonetheless, attempts were made to reduce such biases by ensuring responses were given anonymity and clear instructions were provided. Since this study was conducted at a single center, it is hoped that the findings will help this study become part of future research in a multi-center setting.

### 2.3. Questionnaire

The researchers used the Pittsburgh Sleep Quality Index (PSQI), Beck Depression Inventory (BDI), and Beck Anxiety Inventory (BAI).

The Pittsburgh Sleep Quality Index (PSQI) is a 19-item questionnaire developed to assess sleep quality and patterns over the past month. Each of these questions was scored on a Likert scale ranging from 0 to 3, with higher scores indicating more severe difficulty in sleeping. All the scores were then summed to obtain the global PSQI score, which ranged from 0 to 21. Higher values indicate poorer sleep quality. Additionally, values greater than or equal to five suggest significant sleep disorders [14].

The Beck Depression Inventory (BDI-II) is a test containing twenty-one questions that measures the severity of depression symptoms in the past two weeks. Each item has a score ranging from zero to three, where zero implies “no symptoms” and three means “most serious case”. The BDI-II total score ranges from 0 to 63 for minimal (0–13), mild (14–19), moderate (20–28), and severe (29–63) [15]. Higher scores indicate more severe depressive symptoms.

Lastly, the Beck Anxiety Inventory (BAI) comprises various items that ask about how anxious one has felt during a week, using a scale going from zero to three, with zero indicating absence as no symptom at all; it is rated three when at its most extreme manifestation. Adding these numbers give a total BAI ranging from 0 to 63. Higher overall scores may be suggestive of greater indications of anxiety disorders, i.e., minimal (0–7), mild (8–15), moderate (16–25), and severe (26–63) anxiety [16].

The Pittsburgh Sleep Quality Index (PSQI), Beck Depression Inventory-II (BDI-II), and Beck Anxiety Inventory (BAI) were validated in a way that would render them culturally applicable to the target population. Doctoral prepared nurse and psychometric expert reviewers also made strong recommendations on the use of these instruments after rating them in terms of cultural sensitivity, ease of understanding, and relevance. To further examine the reliability and validity of the instruments, a small sample containing 15 participants was recruited. The results of the pilot study also responded positively to the cultural validity of the instruments. The pilot study resulted in very high internal consistency coefficients, PSQI α = 0.84; BAI α = 0.82; BDI-II α = 0.85, demonstrating the instruments were measuring the intended constructs accurately for the target population.

### 2.4. Data Collection

Prior to data collection, participants signed an informed consent form. The data were collected in January and March in the year 2024 through a questionnaire-based survey. The data collection was conducted by the researchers themselves during routine clinic visits to the hospital. In order to evaluate their sleep quality, depression, and anxiety, the participants filled in the self-administered questionnaires—the Pittsburgh Sleep Quality Index (PSQI), Beck Depression Inventory II (BDI-II), and Beck Anxiety Inventory (BAI). The participants took at least 15 min to answer the questionnaires. Further, demographic data, CKD stage, and history of dialysis as well as comorbidities and medications were captured from the patients’ files. Data privacy measures were extensively maintained throughout this study. The researchers employed a combination of self-report questionnaires and case file review methods to look at sleep quality, mental health, and other clinical variables associated with CKD patients.

### 2.5. Data Analysis

SPSS version 26 was used to analyze the data. Descriptive statistics were used to summarize demographic characteristics, clinical parameters, and mental health outcomes. Chi-square tests were used to assess the association between categorical variables and categorical outcomes. Ordinal logistic regression was used to model the ordinal nature of depression levels and identify factors associated with increased severity. Binary logistic regression was employed to predict the likelihood of severe anxiety or poor sleep quality based on demographic and clinical variables.

### 2.6. Ethical Consideration

Ethical considerations were of paramount importance throughout this study. We obtained approval from the University of Hail Institutional Review Board (H-2023-10) to ensure that the research adhered to ethical guidelines. Maintaining participants’ privacy, anonymity, and confidentiality was the top priority. All data collection procedures were designed to protect participants’ identities. Informed consent was obtained from all participants, and they were free to withdraw from this study at any point without any repercussions.

## 3. Results

Table 1 illustrates the characteristics of CKD patients. The mean age of the participants was (32.67 years), and the majority of them were female (70.4%) and single (58.7%). Participants were mostly at college level (66.5%), with family income ranging 5001–10,000 Saudi Arabian Riyals (SAR) (41.3%). It could be noted that in the majority participants, the cause of CKD was hypertension (52%), the duration of CKD was 3 years (45.8%), hospitalization had occurred three to four times in the past year (62.6%), and Saudi was the nationality (66.5%). Lastly, the duration of dialysis was 3 times a week for 50.8%, with erythropoietin 39.1%, non-smoking status 58.1%, and no surgical history 84.9%.

The Beck Anxiety Inventory (BAI), Beck Depression Inventory-II (BDI-II), and Pittsburgh Sleep Quality Index (PSQI) were utilized to assess anxiety, depression, and sleep quality, respectively. It was observed that almost 66% of the 179 CKD patients were suffering from severe anxiety, whereas about one-third of the patients were moderates (Table 2). Most of patients had minimal depression 40.2%, with those having mild depression accounting for 29.6%, moderate depression 21.2%, and approximately 8.9% with severe depression. On the sleep quality index, more than half (51.4%) who responded reported poor sleep quality.

As shown in Table 3, there was a significant relationship between depression levels and patient characteristics. There was a significant relationship between patients’ gender, marital status, educational level, nationality, and smoking status and the level of depression (*p* < 0.05). On the other hand, there was no significant relationship between patients’ age, family income, causes of CKD, duration of diagnosis, number of hospitalizations, duration of dialysis, medication, and surgical history and the level of depression (*p* > 0.05).

Table 4 illustrates the relationships between patient characteristics, anxiety, and sleep levels. There was a significant difference between the level of anxiety and age, sex, marital status, educational level, and family income (*p* < 0.05). Regarding sleep, there was a significant relationship between the patient’s age, sex, nationality, and the level of sleep (*p* < 0.05). For both anxiety and sleep quality, several sociodemographic characteristics did not demonstrate statistically significant associations. These included causes of CKD, duration of diagnosis, number of hospitalizations, duration of dialysis, medication use (blood pressure medications, phosphate binders, erythropoietin), smoking status, and surgical history.

Ordinal logistic regression was used to explore the predictors of depression among CKD patients. The results indicated that gender, marital status, marital status, educational level (College), nationality, and smoking status were predictors for depression among CKD patients (*p* ˂ 0.05). Female, college, non-Saudi, and smoker patients had less depression than other counterparts. On the other hand, married and divorced patients had more depression than single patients (*p* ˂ 0.05); see Table 5.

Table 6 illustrates the results of the binary logistic regression analysis with anxiety as a dependent variable, and age, gender, marital status, educational level, and family income as independent variables. The results showed that patients aged 26–35 (AOR= 2.792; 95% CI = 1.167–6.679; *p* = 0.021) remained significantly associated with higher anxiety and females (AOR= 0.479; 95% CI = 0.232–0.990; *p* = 0.047) significantly associated with lower anxiety.

Table 7 illustrates the results of the binary logistic regression analysis with poor sleep as a dependent variable, and age, gender, and nationality as independent variables. The results showed that only patients aged 26–35 (AOR= 0.403; 95% CI = 0.188–0.865; *p* = 0.020) remained significantly associated with lower poor sleep scores.

## 4. Discussion

The findings of our study reveal a significant prevalence of anxiety and depression among patients with CKD, with 65.9% experiencing severe anxiety and 40.2% reporting minimal depression. Such indicators are present as research notes that CKD patients are found to have anxiety and depression, which impacts them greatly in day-to-day life in addition to their day-to-day management CKD. The importance of conducting periods of screening for these conditions in routine physical examination is made clear by the confirmation that mental issues negatively affect physical health in this group [17,18]. Moreover, our participants also highlighted high levels of depression and anxiety in relation to the sleep quality they had, and a 52% majority of our participants reported sleep deprivation as well. This is in line with existing studies that have found large depression rates among CKD patients, indicating that these disorders can affect them profoundly [19,20]. The depression and anxiety and sleep disruption patterns are interconnected, and as such, the therapeutic angles for getting better sleep must target the care of patients with CKD. For health specialists, a practical implication of this research is the need to introduce effective screening and treatment of anxiety, depression, and sleep disorders in the management of CKD. From the research data, it can be inferred that prompt response with recognition has favorable outcomes for patients [17,21]. Take, for instance, the chronic tubulointerstitial disease (CTD)-directed model illustrating that CTD-targeted screening triage strategies will have greater attendance rates compared to diabetic kidney disease (DKD). This strongly supports the advantages of structured personalized adapted screening [22]. Additionally, a multi-professional approach should also promote strengthening social and family support of patients with chronic kidney disease since this ensures a better health outcome physically or even mentally [19]. In comparison, our results on the occurrence of anxiety and depression among CKD patients is also supported by other studies with varying rates of occurrence, such as in Hedayati et al. [23], who reported 21%. These discrepancies may arise from differences in study populations, methodologies, and the specific CKD stages examined. These gaps must be addressed, as they will inform the design of screening and intervention strategies that meet the needs of distinct patient populations [17,23]. The high levels of anxiety and depression and poor sleep quality found in patients with CKD indicate that screening interventions for their mental health is important and should be carried out urgently. Once this practice is integrated into routine care, practitioners can have a major impact on improvements to the quality of life and health of people with chronic kidney disease.

The findings of the current study indicate a significant correlation between depression scores and various sociodemographic factors among patients with CKD. The variables determined to be influential included gender, marital status, educational level, nationality, and smoking status. This is fair given the available literature that consistently demonstrates the link between sociodemographic characteristics and mental health of persons with CKD. In particular, it has been observed that the people with a low education level and those not working suffer more from anxiety and depression, which implies that their economic status is crucial to their psychological well-being [24]. Female patients and single patients have been found to suffer more depressive symptoms compared to married ones, which suggests that marital status and gender must be included among the determinants of mental health in CKD patients [25]. However, the current study results differ in that previous studies have shown no significant relationships between clinical characteristics such as age, family income, and the period of time on dialysis and levels of depression. One study found that cognitive impairment was associated with depressive symptoms among patients with advanced CKD, hinting that it is a multi-faceted phenomenon caused by different clinical mechanisms and the patient’s mental state [26]. Such variation may be a result of the peculiar sociodemographic features of the study group, who were more mentally healthy than clinically healthy. The implications of these insights are that depression in CKD patients is not singular in nature and in its management; sociodemographic variables have to be factored in. 

Anxiety is another prevalent mental health issue among CKD patients, with studies indicating varying prevalence rates. It was stated that the level of reporting anxiety in CKD patients was 50.4%, which is still lower than other studies that reported higher than 71% levels of reporting anxiety [27]. This divergence in reporting could possibly be due to changes in gender composition and study methods in these studies. According to previous research, the prevalence rates for depression in CKD patients were found to be far higher, indicating that this condition is a widespread mental disorder among this group of patients [10]. This research also supports this theory, concluding that social variables and hypertension have been found to be strongly connected to high levels of depression. In addition to this, it was stated that hemodialysis patients are inclined towards a depressed state more often, so it deserves an introduction of more precise mental health-related programs for this category of individuals [28]. Likewise, patients with depression and anxiety in patients with CKD reported increased incidence of sleep disturbances. Based on evidence from numerous studies, it has been determined that sleep problems significantly compound depression, thus creating a vicious circle that affects physical and mental health [29]. Resolving sleep problems might prove to be beneficial towards ensuring better mental health for the CKD patients. More specifically, the sociodemographic variables of the patients are of paramount importance. Other studies have indicated that a lower educational status coupled with unemployment and being single are significant determinants of greater anxiety and depression rates among populations [24,25]. One such reasoning is that a multi-approach strategy is more likely to work by addressing clinical issues while at the same time improving economic conditions and other forms of vulnerability. With such evidence available, practitioners are advised to consider an all-rounded view of psychological health in patients with CKD. There is an urgent need to expand the scope of the intervention to incorporate sociodemographic variables such as age, gender, marital status, and socioeconomic strata in intervention design. Some of these actionable recommendations could include depression support programs, ameliorating access to mental healthcare, and enabling social support to help patients feel less alone and hopeless.

The current study’s findings reveal statistically significant differences in anxiety levels based on various sociodemographic factors, including age, sex, marital status, education level, and family income. This congruence has also been reported in the existing literature, where it was noted that sociodemographic attributes are of paramount importance for patients with CKD as it relates to mental health. For example, it has been suggested that there is a need for routine assessment of anxiety and depressive symptoms amongst CKD patients [10] emphasizing that socioeconomic factors such as educational level and income conditions are of great significance in the therapeutic outcomes of mental disorders [24]. In the same vein, Huang et al. also pointed marital status and education as determinants of anxiety levels in CKD patients, contributing more to the significance of these sociodemographic characteristics to mental health [25]. A fair correlation analysis involving age, sex, and nationality in relation to sleep quality has been brought out that tends to corroborate the assertion that elderly women and patients generally have poor sleep habits. Both age-related physiology changes and gender-related factors explain this observation [30]. Although the present study found that sociodemographic characteristics are significant predictors of anxiety and quality of sleep, it also observed that there are no significant relationships between psychological outcomes and clinical factors such as duration on dialysis and number of hospital visits. This finding is at odds with some of the literature suggesting that clinical characteristics, such as severity of CKD and treatment rest, have an effect on anxiety and depression level [25]. The fact that clinical characteristics are important but sociodemographic characteristics are more important in explaining anxiety and quality of sleep among patients with CKD helps explain this discrepancy. The importance of integrating sociodemographic and clinical models when addressing issues of anxiety and sleep disturbances in CKD patients was also emphasized. Future research should aim to explore these relationships further so as to inform on the best interventions to deal with the psychological problems of patients with CKD and, over and above, improve their overall health and clinical outcomes.

The results of the present study show that in patients with CKD, sociodemographic components such as gender, age, marital status, nationality, and smoking have significant and specific effects on depression. Specifically, depression is more prevalent among divorced or married patients than single ones. The cited studies confirm the presence of such a phenomenon as the relationship of demographic characteristics with depression among patients with CKD. For instance, low educational level and being married also contribute to the high depression in CKD patients, indicating that the level of depression is determined by social and cultural factors [25]. Moreover, it was determined that different sociodemographic variables such as nationality or social relations influence the mental health variables, which goes to show that sociocultural factors are pertinent in classifying mental illness in this group [31]. In conditions of CKD, it is typical to consider that probes of depression in women are higher than in men, and this is directly related to women, but some researchers refute this point, saying that women suffer less depression than men. However, variation in gender differences of depression varies with respect to sample characteristics and sites, and the differences may be due to the diverse population studied [32]. These comments are consistent with previous research, which notes the strong need to take into account smoking status in gender differences in depression in groups where there are strong gender differences in smoking [33].

The present results corroborate previous findings that show more anxiety among younger CKD patients. This finding can help in explaining the motivation for undertaking the current study, particularly the fact that younger individuals with CKD tend to display higher anxiety levels, as was stated earlier. These patients do tend to have higher anxiety levels due to the various pressures due to long-term illness; however, age is marked as a more prominent risk factor. The female patients in the current study were found to be less anxious than the men. Such an observation is not frequently reported in the literature, where women are generally assumed to demonstrate more anxiety and depression at large, notably in chronic illness settings. Nonetheless, the source from the earlier study does not seem to connect gender and anxiety amongst the CKD patients and has a correlative connection between depression and mortality instead [34]. Thus, there is a need for relevant literature that examines gender differences in anxiety levels among CKD patients. Furthermore, the sociodemographic characteristics and their influence on anxiety suggest that these factors need to be evaluated in processes involving CKD patients. Targeted strategies channeling anxiety in patients considering their age, sex, and other demography variables can be formulated by healthcare providers. Such an intervention approach would help in ameliorating the mental health of CKD patients through innovative strategies purposefully focusing on the experience of the relevant population.

The present study’s findings indicate that younger patients, specifically those aged 26–35 years, experience a significantly lower prevalence of poor sleep scores compared to older CKD patients. The issues surrounding sleep quality in the existing literature also focus on age factors, with younger adults tending to have better sleep as compared to older adults who are prone to having a plethora of other age related issues. Shifts in sleep patterns were noted more among older individuals, while sleep disorders were more common in elderly populations, which were further corroborated in further programs [35]. Distressed circadian cycles and other steadily increasing comorbid factors among the older population may explain the preceding trend. Surprisingly, the current research, while remaining consistent with some past studies that did not find any significant relationships between gender differences and other explanatory variables in reply to sleep quality in CKD patients, found that gender and nationality were not particularly indicative. By and large, the results of this study demonstrate once again the necessity of enhancing breastfeeding support as a disability equalization strategy, especially in much younger CKD patients residing in the city. The overall well-being and quality of life of CKD patients is hugely impacted by their sleep; therefore, it is essential to address sleeping issues, as enhanced sleep quality leads to lower chances of illness and death. It was stressed that sleep quality can greatly affect the health outcomes of people suffering from chronic illness; therefore, healthcare providers need to investigate clinics related to sleep in the context of CKD management [36]. Hence, additional studies are needed to investigate the self-management strategies adopted by patients with CKD in relation to their sleeping behaviors, particularly those focused on enhancing sleep performance, which will subsequently lead to better overall health.

## 5. Implications to Mental Health

This study may have strong implications, as there is a gap in individual mental health assessment and interventions amongst CKD patients. Also, the level of severe anxiety and depression is high in those with CKD, meaning that more attention is needed to their mental health since it may relate to their quality of life and clinical outcomes. Considering more than half of the respondents expressed such sleep disturbances being an important factor that needs to be given due diligence, the results recommend that sociodemographic factors—gender, marital status, education level, nationality, and currently smoking cigarettes—are all important predictors of depression and anxiety among CKD patients. For example, the database reveals that married and divorced respondents were the most depressed, while younger patients suffered the most anxiety. The findings of this research are consistent with the literature that focuses on the role of sociodemographic crystal variables in the mental health assessment of CKD patients. Also, the provided data about the relationships between anxiety, depression, and sleep quality further point out the comorbidity of the mentioned medical conditions. Sleep disturbances are known to increase both anxiety and depression, which forms a cycle that can impair treatment effectiveness and worsen patient outcomes. For that reason, alternate medicine for sleep problems in patients with CKD should be given high priority because it may reduce anxiety and depression in patients. In this case, the current study demonstrates the evidence of the need for regular mental health assessment of patients with CKD, as this would allow for the implementation of timely and more effective treatment. It would also be pertinent to study CKD patients suffering from anxiety and depression and apply their relevant treatment strategies such as cognitive behavioral therapy or medications aimed at these specific disorders. Furthermore, it should be stressed that factors such as demographic characteristics, mental health, and sleep structure were explained poorly in the patients with continuous kidney disease and further studies are needed to clarify this point for developing future interventions aimed at enhancing the quality of life of these patients.

## 6. Study Limitations

This study had several limitations. The cross-sectional design precludes the establishment of causal relationships between variables. Additionally, the use of convenience sampling in a single hospital limited the generalizability of the findings. Furthermore, self-report questionnaires may be susceptible to recall bias and social desirability effects. Future research using longitudinal designs and more diverse samples is needed to confirm these findings and explore causal pathways. Additionally, investigating the potential influence of medications and social support on the mental health of patients with CKD would be valuable.

## 7. Conclusions

Given the high frequency of anxiety, depression, and even insomnia, which were high in the CKD population, the results of this study point towards carrying out routine mental assessments as a priority. If nearly two patients out of three are reporting high levels of anxiety and a significant percentage of patients present with depressive symptoms and insomnia, it automatically points to the need for healthcare providers to take an active stance on these challenges in relation to mental health. Possible options that may be used for this purpose include but are not limited to regular mental health checks, integrated clinical care development, patient education, targeted campaigns or interventions, and even community approaches. There is also a special aspect regarding affected patients that has to be addressed, namely, the implications for general health policies, meaning there is a justified belief of the need for greater financial resources for interdisciplinary approaches to the development of management of CKD guidelines and policies. In the future, research efforts need to be directed to longitudinal studies in order to try to clarify the causal links between population characteristics and mental health disorders and to assess the helpfulness of interventions to such mental disorders’ outcomes.

## Figures and Tables

**Table 1:** Sociodemographic characteristics of chronic kidney disease patients. n = 179.

Variable	Characteristics	n	%
Age	Mean ± SD (32.67 ± 10.54 years) Range: 22–66 years		
	25 or less	77	43.000
	26–35	60	33.500
	36 or more	42	23.500
Gender	Male	53	29.600
	Female	126	70.400
Marital status	Single	105	58.700
	Married	70	39.100
	Divorced	4	2.200
Educational attainment	high school	32	17.900
	College	119	66.500
	master	28	15.600
Family income (in terms of Saudi Arabian Riyals [SAR])	5000 SAR or less	44	24.600
	5001–10,000 SAR	74	41.300
	10,001 SAR or more	61	34.100
Nationality	Saudi	119	66.500
	Non-Saudi	60	33.500
Causes of CKD	Diabetes Mellitus	63	35.200
	Hypertension	93	52.000
	Others	23	12.800
Duration of diagnosis	2 years or less	51	28.500
	3 years	82	45.800
	4 years or more	46	25.700
Number of hospitalizations (in a year)	1–2	12	6.700
	3–4	112	62.600
	5 or more	54	30.200
Duration of dialysis (in a week)	2 or less	38	21.200
	3	91	50.800
	4 or more	50	27.900
Medication	Blood pressure medication	42	23.500
	Phosphate	67	37.400
	Erythropoietin	70	39.100
Smoking	No	104	58.100
	Yes	75	41.900
Surgical history	No	152	84.900
	Yes	27	15.100

**Table 2:** Prevalence of sleep disorder, anxiety, and depression among 179 CKD patients.

Variable	Level	Frequency	Percentage
Anxiety	Moderate	61	34.1
	Severe	118	65.9
Depression	Minimal	72	40.2
	Mild	53	29.6
	Moderate	38	21.2
	Severe	16	8.9
Sleep	Normal	87	48.6
	Poor sleep	92	51.4

**Table 3:** Association between sociodemographic characteristics and the level of depression among 179 CKD patients.

Variable	Characteristics	n	Minimal	Mild	Moderate	Severe	*p*
Age	25 or less	77	33 (42.9)	16 (20.8)	19 (24.7)	9 (11.7)	0.101
	26–35	60	28 (46.7)	20 (33.3)	8 (13.3)	4 (6.7)	
	36 or more	42	11 (26.2)	17 (40.5)	11 (26.2)	3 (7.1)	
Gender	Male	53	12 (22.6)	26 (49.1)	12 (22.6)	3 (5.7)	<0.001
	Female	126	60 (47.6)	27 (21.4)	26 (20.6)	13 (10.3)	
Marital status	Single	105	48 (45.7)	28 (26.7)	20 (19.0)	9 (8.6)	0.020
	Married	70	24 (34.3)	25 (35.7)	16 (22.9)	5 (7.1)	
	Divorced	4	0 (0.0)	0 (0.0)	2 (50.0)	2 (50.0)	
Educational level	High school	32	6 (18.8)	16 (50.0)	6 (18.8)	4 (12.5)	0.026
	College	119	57 (47.9)	26 (21.8)	26 (21.8)	10 (8.4)	
	Master	28	9 (32.1)	11 (39.3)	6 (21.4)	2 (7.1)	
Family income	5000 or less	44	17 (38.6)	16 (36.4)	9 (20.5)	2 (4.5)	0.080
	5001–10,000	74	35 (47.3)	20 (27.0)	16 (21.6)	3 (4.1)	
	10,001 or more	61	20 (32.8)	17 (27.9)	13 (21.3)	11 (18.0)	
Nationality	Saudi	119	47 (39.5)	27 (22.7)	29 (24.4)	16 (13.4)	0.001
	Non-Saudi	60	25 (41.7)	26 (43.3)	9 (15.0)	0 (0.0)	
Causes of CKD	DM	63	25 (39.7)	17 (27.0)	15 (23.8)	6 (9.5)	0.670
	HTN	93	40 (43.0)	26 (28.0)	20 (21.5)	7 (7.5)	
	Others	23	7 (30.4)	10 (43.5)	3 (13.0)	3 (13.0)	
Duration of diagnosis	2 or less	51	19 (37.3)	16 (31.4)	11 (21.6)	5 (9.8)	0.498
	3	82	34 (41.5)	28 (34.1)	13 (15.9)	7 (8.5)	
	4 or more	46	19 (41.3)	9 (19.6)	14 (30.4)	4 (8.7)	
Number of hospitalizations	1–2	12	5 (41.7)	2 (16.7)	2 (16.7)	3 (25.0)	0.520
	3–4	112	47 (42.0)	33 (29.5)	23 (20.5)	9 (8.0)	
	5 or more	54	20 (36.4)	18 (32.7)	13 (23.6)	4 (7.3)	
Duration of dialysis	2 or less	38	14 (36.8)	10 (26.3)	11 (28.9)	3 (7.9)	0.933
	3	91	37 (40.7)	28 (30.8)	18 (19.8)	8 (8.8)	
	4 or more	50	21 (42.0)	15 (30.0)	9 (18.0)	5 (10.0)	
Medication	BP medication	42	16 (38.1)	10 (23.8)	12 (28.6)	4 (9.5)	0.520
	Phosphate	67	23 (34.3)	24 (35.8)	15 (22.4)	5 (7.5)	
	Erythropoietin	70	33 (47.1)	19 (27.1)	11 (15.7)	7 (10.0)	
Smoking	No	104	41 (39.4)	25 (24.0)	22 (21.2)	16 (15.4)	0.003
	Yes	75	31 (41.3)	28 (37.3)	16 (21.3)	0 (0.0)	
Surgical history	No	152	62 (40.8)	44 (28.9)	34 (22.4)	12 (7.9)	0.557
	Yes	27	10 (37.0)	9 (33.3)	4 (14.8)	4 (14.8)	

Note: CKD—chronic kidney disease; DM—diabetes mellitus; HTN—hypertension.

**Table 4:** Association between sociodemographic characteristics and levels of anxiety and sleep among 179 chronic kidney disease patients.

Variables		n	Moderate	Severe	*p*	Normal Sleep	Poor Sleep	*p*
Age	25 or less	77	35 (45.5)	42 (54.5)	<0.001	47 (61.0)	30 (39.0)	0.009
	26–35	60	23 (38.3)	37 (61.7)		21 (35.0)	39 (65.0)	
	36 or more	42	3 (7.1)	39 (92.9)		19 (45.2)	23 (54.8)	
Gender	Male	53	9 (17.0)	44 (83.0)	0.002	19 (35.8)	34 (64.2)	0.027
	Female	126	52 (41.3)	74 (58.7)		68 (54.0)	58 (46.0)	
Marital status	Single	105	45 (42.9)	60 (57.1)	0.008	54 (51.4)	51 (48.6)	0.305
	Married	70	16 (22.9)	54 (77.1)		30 (42.9)	40 (57.1)	
	Divorced	4	0 (0.0)	4 (100.0)		3 (75.0)	1 (25.0)	
Educational level	high school	32	6 (18.8)	26 (81.3)	<0.001	21 (65.6)	11 (34.4)	0.103
	College	119	53 (44.5)	66 (55.5)		53 (44.5)	66 (55.5)	
	Master	28	2 (7.1)	26 (92.9)		13 (46.4)	15 (53.6)	
Family income	5000 or less	44	12 (27.3)	32 (72.7)	0.007	27 (61.4)	17 (38.6)	0.121
	5001–10,000	74	35 (47.3)	39 (52.7)		31 (41.9)	43 (58.1)	
	10,001 or more	61	14 (23.0)	47 (77.0)		29 (47.5)	32 (52.5)	
Nationality	Saudi	119	43 (36.1)	76 (63.9)	0.259	65 (54.6)	54 (45.4)	0.023
	Non-Saudi	60	18 (30.0)	42 (70.0)		22 (36.7)	38 (63.3)	
Causes of CKD	DM	63	23 (36.5)	40 (63.5)	0.405	31 (49.2)	32 (50.8)	0.910
	HTN	93	33 (35.5)	60 (64.5)		44 (47.3)	49 (52.7)	
	Others	23	5 (21.7)	18 (78.3)		12 (52.2)	11 (47.8)	
Duration of Diagnosis	2 or less	51	16 (31.4)	35 (68.6)	0.845	25 (49.0)	26 (51.0)	0.963
	3	82	28 (34.1)	54 (65.9)		39 (47.6)	43 (52.4)	
	4 or more	46	17 (37.0)	29 (63.0)		23 (50.0)	23 (50.0)	
Number of hospitalizations	1–2	12	3 (25.0)	9 (75.0)	0.734	5 (41.7)	7 (58.3)	0.712
	3–4	112	40 (35.7)	72 (64.3)		53 (47.3)	59 (52.7)	
	5 or more	54	18 (32.7)	37 (67.3)		29 (52.7)	26 (47.3)	
Duration of dialysis	2 or less	38	13 (34.2)	25 (65.8)	0.936	17 (44.7)	21 (55.3)	0.864
	3	91	30 (33.0)	61 (67.0)		45 (49.5)	46 (50.5)	
	4 or more	50	18 (36.0)	32 (64.0)		25 (50.0)	25 (50.0)	
Medication	BP medication	42	13 (31.0)	29 (69.0)	0.872	20 (47.6)	22 (52.4)	0.956
	Phosphate	67	24 (35.8)	43 (64.2)		32 (47.8)	35 (52.2)	
	Erythropoietin	70	24 (34.3)	46 (65.7)		35 (50.0)	35 (50.0)	
Smoking	No	104	37 (35.6)	67 (64.4)	0.369	54 (51.9)	50 (48.1)	0.295
	Yes	75	24 (32.0)	51 (68.0)		33 (44.0)	42 (56.0)	
Surgical history	No	152	54 (35.5)	98 (64.5)	0.229	73 (48.0)	79 (52.0)	0.714
	Yes	27	7 (25.9)	20 (74.1)		14 (51.9)	13 (48.1)	

Note: CKD—chronic kidney disease; DM—diabetes mellitus; HTN—hypertension.

**Table 5:** Ordinal logistic regression of factors affecting depression among 179 chronic kidney disease patients.

Factor		B	S.E.	Wald	AOR	95% CI for Exp (B)	Sig.
Threshold 1		−1.785	0.451	15.687	0.168	−2.668–−0.902	0.000
Threshold 2		−0.398	0.430	0.855	0.672	−1.241–0.445	0.355
Threshold 3		1.271	0.469	7.349	3.564	0.352–2.190	0.007
Gender	Male	Reference					
	Female	−0.728	0.321	5.146	0.483	−1.358–−0.099	0.023
Marital status	Single	Reference					
	Married	0.719	0.337	4.558	2.052	0.059–1.379	0.033
	Divorce	3.392	1.068	10.082	29.733	1.298–5.486	0.001
Educational level	High school	Reference					
	College	−0.761	0.381	3.996	0.467	−1.507–−0.015	0.046
	Master	−0.465	0.517	0.807	0.628	−1.478–0.549	0.369
Nationality	Saudi	Reference					
	Non-Saudi	−0.789	0.346	5.207	0.454	−1.466–−0.111	0.023
Smoking	No	Reference					
	Yes	−0.781	0.298	6.886	0.458	−1.365–−0.198	0.009

B—coefficient of predictor variables; S.E.—standard error; AOR—adjusted odds ratio; CI—confidence interval.

**Table 6:** Binary logistic regression of factors affecting anxiety among 179 chronic kidney disease patients.

Factor		B	S.E.	Wald	AOR	95% CI for Exp (B)	Sig.
Age	25 or less	Reference					
	26–35	1.027	0.445	5.321	2.792	1.167–6.679	0.021
	36 or more	0.541	0.478	1.280	1.718	0.673–4.385	0.258
Gender	Male	Reference					
	Female	−0.736	0.370	3.948	0.479	0.232–0.990	0.047
Marital status	Single	Reference					
	Married	−0.150	0.383	0.154	0.860	0.406–1.823	0.695
	Divorce	−1.431	1.211	1.396	0.239	0.022–2.566	0.237
Educational level	High school	Reference					
	College	0.623	0.480	1.684	1.864	0.728–4.775	0.194
	Master	0.295	0.648	0.207	1.343	0.377–4.787	0.649
Family income	5000 or less	Reference					
	5001–10,000	0.335	0.464	0.522	1.399	0.563–3.473	0.470
	10,001 or more	0.331	0.446	0.552	1.392	0.581	0.458

B—coefficient of predictor variables; S.E.—standard error; AOR—adjusted odds ratio; CI—confidence interval.

**Table 7:** Binary logistic regression of factors affecting poor sleep among 179 chronic kidney disease patients.

Factor		B	S.E.	Wald	AOR	95% CI for Exp (B)	Sig.
Age	25 or less	Reference					
	26–35	−0.909	0.390	5.444	0.403	0.188–0.865	0.020
	36 or more	−0.361	0.439	0.676	0.697	0.295–1.648	0.411
Gender	Male	Reference					
	Female	0.665	0.350	3.602	1.944	0.979–3.861	0.058
Nationality	Saudi	Reference					
	Non-Saudi	−0.350	0.378	0.859	0.704	0.336–1.478	0.354

Note: B—coefficient of predictor variables; S.E.—standard error; AOR—adjusted odds ratio; CI—confidence interval.

## Data Availability

The datasets generated in this study are available from the corresponding author upon request.
